# Residues 315 and 369 in HN Protein Contribute to the Thermostability of Newcastle Disease Virus

**DOI:** 10.3389/fmicb.2020.560482

**Published:** 2020-09-22

**Authors:** Baoyang Ruan, Xiaorong Zhang, Chengcheng Zhang, Pengyu Du, Chengcheng Meng, Mengjiao Guo, Yantao Wu, Yongzhong Cao

**Affiliations:** ^1^College of Veterinary Medicine, Yangzhou University, Yangzhou, China; ^2^The Joint International Research Laboratory of Agriculture & Agri-Product Safety, Yangzhou University, Yangzhou, China

**Keywords:** Newcastle disease, thermostability, HN gene, mutations, chimeric viruses

## Abstract

Thermostable Newcastle disease virus (NDV) vaccines have been widely used in areas where a “cold-chain” is not reliable. However, the molecular mechanism of NDV thermostability remains poorly understood. In this work, we constructed chimeric viruses by exchanging viral fusion (F) and/or hemagglutinin-neuraminidase (HN) genes between the heat-resistant strain HR09 and thermolabile strain La Sota utilizing a reverse genetic system. The results showed that only chimeras with HN derived from the thermostable virus exhibited a thermostable phenotype at 56°C. The hemagglutinin (HA) and neuraminidase (NA) activities of chimeras with HN derived from the HR09 strain were more thermostable than those containing HN from the La Sota strain. Then, we used molecular dynamics simulation at different temperatures (310 K and 330 K) to measure the HN protein of the La Sota strain. The conformation of an amino acid region (residues 315–375) was observed to fluctuate. Sequence alignment of the HN protein revealed that residues 315, 329, and 369 in the La Sota strain and thermostable strains differed. Whether the three amino acid substitutions affected viral thermostability was investigated. Three mutant viruses based on the thermolabile strain were generated by substituting one, two or three amino acids at positions 315, 369, and 329 in the HN protein. In comparison with the parental virus, the mutant viruses containing mutations S315P and I369V possessed higher thermostablity and HA titers, NA and fusion activities. Taken together, these data indicate that the HN gene of NDV is a major determinant of thermostability, and residues 315 and 369 have important effects on viral thermostability.

## Introduction

Newcastle disease virus (NDV) is a highly contagious and important cause of loss in the poultry industry (Sinkovics and Horvath, 2000; Miller et al., 2010). It is a member of the avian paramyxoviruses (APMV) and an enveloped virus that contains a single stranded, negative sense RNA genome. The NDV genome contains six genes encoding the nucleoprotein (NP), phosphoprotein (P), matrix protein (M), fusion protein (F), hemagglutinin-neuraminidase protein (HN) and large RNA polymerase (L) (Dimitrov et al., 2019). Infection of host cells by NDV is accomplished by the interaction of two surface glycoproteins, F and HN (Gravel and Morrison, 2003; Rangaswamy et al., 2017). The F protein directs the viral fusion activity and is supposed to play a major role in determining NDV virulence (Samal et al., 2013). HN is responsible for activating the F protein, which mediates the fusion of the viral envelope with the host cell membrane (Mirza and Iorio, 2013). Furthermore, HN is a multifunctional molecule that contains a receptor binding site for sialic acid and has neuraminidase (NA) activity to hydrolyze sialic acid from progeny virions to prevent viral self-aggregation (Porotto et al., 2006).

Most NDV strains are thermolabile and lose infectivity upon exposure to 50–55°C for 30 min. However, heat-resistant strains (such as V4) can maintain their hemagglutinin (HA) activity and infectious ability at 56°C for at least 30 min (Simmons, 1967). Previous studies showed that most thermostable strains belong to genotype I and possess low pathogenicity to chickens, and the thermostable vaccines have been widely used in areas where a “cold-chain” is not reliable (Mahmood et al., 2014; Dimitrov et al., 2017). Previously, the F, HN and P genes have been studied regarding their relations with virus thermostability by constructing chimeric viruses based on V4 (Wen et al., 2016; Zhao et al., 2018; Liu et al., 2019). However, thermostable and virulent strains (such as AF2240-I) belonging to genotype VIII have many differences in molecular and antigenic characteristics with V4 (Murulitharan et al., 2013). Currently, similar thermostability research based on virulent and thermostable strains has not been performed. The underlying correlations between the viral envelope glycoproteins and thermostability remain unclear.

For the molecular characterization of thermostable NDV, the genome of several thermostable strains have been sequenced. By analyzing the sequences of different thermostable strains, Yusoff et al. (1996) found that deletion of amino acid Arg (403) of the HN protein may influence the thermostability of NDV. Nevertheless, Kattenbelt et al. (2006) had attempted to find this alteration in thermostable strains but were not successful. Uthrakumar et al. (2013) found four and eleven amino acid substitutions in the HN and L genes, respectively, by comparison with the sequences of progenitor and thermostable stains and proposed that these changes in the amino acid sequences might contribute to the thermostability of NDV. In addition, Omony et al. (2016) noted there is a higher proportion of Ile, Leu, Val, and Arg at the stalk and globular regions of the HN protein in thermostable strains. In addition, substitution of amino acids at the conserved stalk spike may affect the NA activity of the globular domain which attaches sialic acid receptors. However, the molecular mechanism of NDV thermostability is still poorly understood.

In this study, we generated chimeric NDVs by exchanging viral F and/or HN genes between the heat-resistant strain HR09 (genotype VIII) and thermolabile strain La Sota. The differences in thermostability among these chimeric viruses were evaluated to identify thermostable determinants of NDV. Our results showed that the HN gene is a crucial determinant for NDV thermostability. Furthermore, we further performed a molecular dynamic simulation to analyze the stability of the HN protein structure at different temperatures and compared the amino acid sequences of thermostable and thermolabile viruses. We then investigated the influence of amino acid substitutions at positions 315, 369, and 329 on viral thermostability. The results showed that the mutations of S315P and I369V in the HN protein significantly enhanced the viral thermostability.

## Materials and Methods

### Cells, Viruses, and Plasmids

The NDV heat-resistant strain HR09 was isolated from chickens and stored in our laboratory (Cao et al., 2017). The NDV thermolabile strain La Sota was provided by the Animal Infectious Disease Laboratory of Yangzhou University. BSR T7/5 cells stably expressing T7 RNA polymerase were provided by Harbin Veterinary Institute. The cell lines were maintained in DMEM (Gibco) supplemented with 10% fetal bovine serum (FBS), 100 U/mL of penicillin and 100 μg/mL of streptomycin. Supporting plasmids (NP, P, and L) were constructed successfully and stored in our laboratory. The TVT7R (0.0) plasmid was a gift from Professor L. Andrew Ball (University of Alabama, United States).

### Construction of Infectious cDNA Clones of Chimeras

The full-length infectious cDNA clones (ICs) of the NDV heat-resistant strain HR09 and thermolabile strain La Sota, named cHR and cLa, respectively, were generated in plasmid TVT-7R (0.0) (Johnson et al., 2000) from six fragments that were amplified by PCR using high-fidelity DNA polymerase (New England BioLabs, United States) as described in our previous research (Ruan et al., 2020).

**FIGURE 1:**
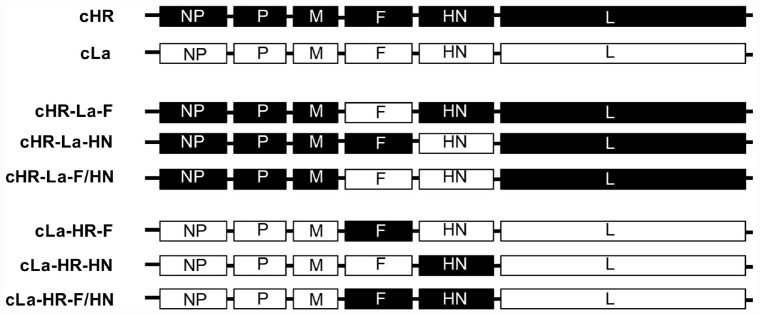
Schematic representation of the construction of chimeric NDVs. Black and white bars indicate the genes of the HR09 and La Sota strain, respectively. The corresponding gene fragments were fused by using the HiFi DNA assembly cloning method.

Three chimeric ICs, cHR-La-F, cHR-La-HN, and cHR-La-F/HN, were constructed by replacing the open reading frames (ORFs) of the F and/or HN genes in the cHR strain with the counterpart fragments from the La Sota strain (Figure 1). Briefly, the ORFs of the F and/or HN genes were amplified by PCR and then inserted into the corresponding sites in full-length ICs of La Sota according to the HiFi DNA assembly cloning method (New England BioLabs, United States). Similarly, ICs of the chimeric viruses cLa-HR-F, cLa-HR-HN and cLa-HR-F/HN were generated by replacing the ORFs of the F and/or HN genes in cLa with the corresponding fragments from cHR (Figure 1).

### Sequence Alignment and Homology Modeling

To analyze the amino acid sequence differences in the HN proteins of the thermostable NDV strains and La Sota strain, the HN gene sequences of currently available thermostable NDV strains were retrieved from GenBank. The amino acid sequences were aligned by Clustal W, and the results were analyzed by BioEdit software.

To further investigate the possible mechanism of improved viral thermostability, the 3D structure of the HN protein was modeled by SWISS-MODEL software online^1^. The HN protein structure was visualized and analyzed with VMD software (Softpedia, United States).

### Molecular Dynamics Simulations

The crystal structures of the HN protein of the La Sota strain were obtained from the Protein Data Bank (PDB ID code: 3t1e.1.A) and analyzed by molecular dynamics (MD) simulation, and potentially flexible regions in the HN protein were identified. The MD simulations were carried out by NAMD software (Phillips et al., 2005) with a CHARMM27 force filed (MacKerell et al., 1998). NA^+^ and Cl^–^ were added to neutralize the system. The HN protein was solvated in a cubic box consisting of the TIP3P water model, and the distance of protein atoms from the wall was more than 10 Å. The whole system was minimized using the descent method (10000 steps). A time step of 50 ps was used to balance the conformation before performing the MD simulation. By calculating the root mean square deviation (RMSD) and root mean square fluctuation (RMSF) values for backbone atoms at temperatures of 310 K (approximately 37°C) and 330 K (approximately 57°C), respectively, thermally sensitive regions of the HN protein were identified.

### Amino Acid Mutations in HN Protein and Construction of Infectious cDNA Clones

To investigate whether the three amino acid substitutions at the thermally sensitive regions of the HN protein affect viral thermostability, the full-length ICs of thermolabile cHR-La-HN were used as the backbone, and chimeric ICs containing substitution of single, double or triple amino acids at positions 315, 369, and 329 were constructed. Briefly, the mutations of S315P, I369V, and V329A generated on plasmids pLa-HN using PCR-based site-directed mutagenesis with three pairs of primers (Table 1). Finally, the three full-length ICs were generated by the HiFi DNA assembly cloning method. The mutated full-length ICs containing S315P, S315P&I369V and S315P&I369V&V329A substitutions were designated cHR-La-HN-A, cHR-La-HN-B, and cHR-La-HN-C, respectively, according to the mutation site (Figure 6B).

**TABLE 1:** Primer sequences of construction for mutant NDVs.

Primer	Sequence (5′-3′)	Location^a^
La-HN-315-F	ACAGCCGCGTATGGTTCcCAGTCTACGGAGG	926–956 nt
La-HN-315-R	gGAACCATACGCGGCTGTCAATAAAAGATCCA	912–943 nt
La-HN-369-F	GACGGTTTGGTGGGAAACGAgTACAGCAGGCTAT	1085–1118 nt
La-HN-369-R	cTCGTTTCCCACCAAACCGTCCAGGCTTATACGA	1072–1105 nt
La-HN-329-F	ATTCACCCAGTGACACTGcACAGGAAGGGAAA	968–999 nt
La-HN-329-R	gCAGTGTCACTGGGTGAATTGGGTTTTAACC	956–986 nt

*^*a*^Full-length antigenomic cDNA of the LaSota strain. Lowercase letters indicate the mutant nucleotides.*

### Rescuing and Propagation of Viruses

Rescuing of chimeric and mutant viruses was performed by cotransfecting the full-length ICs with three supporting plasmids (NP, P, and L) into BSR T7/5 cell lines using Lipofectamine 3000 (Invitrogen, United States), as previously described (Hu et al., 2017). Briefly, a final concentration of 10% allantoic fluid was added to the BSR T7/5 cells at 12 h post-transfection. The rescued viruses were propagated by inoculating 400 μL of cell lysate into the allantoic cavity of 10-day-old SPF chicken embryos. Four days after inoculation, the infected allantoic fluids were harvested, and the rescued viruses were examined by HA assay using 1% chicken red blood cells. All generated viruses were passaged in 10-day-old SPF chicken embryos five times, and the sequences of the F and HN genes were confirmed by sequencing.

### Evaluation of Biological Characteristics

The pathogenicity of the rescued NDVs in 10-day-old SPF chicken embryos was determined by conducting the mean death time (MDT) assay (Alexander, 2000). The titers of NDV were determined by performing the HA test and 50% egg infectious dose (EID_50_) assay (Edwards, 2007). The growth kinetics of NDVs were determined in chicken embryo fibroblasts (CEFs) in the presence of exogenous protease. Triplicates of 6-well plates seeded with approximately 10^6^ CEFs per well were infected at a multiplicity of infection (MOI) of 0.01 for 2 h, then washed three times with phosphate-buffer saline (PBS), and overlaid with medium containing 2% FBS and 2 μg/mL TPCK-treated trypsin (Sigma, United States). The infected cell supernatants were harvested at the indicated time points, and the virus was titrated using the 50% tissue culture infective dose (TCID_50_) assay according to the method of Reed and Muench (1938).

### Thermostability Test

One milliliter of allantoic fluids infected with NDV were submerged into a water bath at 56°C, and transferred to an ice-cold water bath at the indicated time points to stop the heat treatment (Jeong et al., 2013). The infectivity and HA activity of these heat-treated viruses were determined by titration by performing the EID_50_ assay and the HA test, respectively. HA assay was performed by micro-hemagglutination procedure using microtiter method. Briefly, the viruses were two-fold serially diluted with PBS, and then equal volumes of fresh 1% (V/V) chicken red blood cells were added and incubated at 37°C for 30 min. The HA titers of the viruses were determined as the highest dilution giving 100% agglutination. The decreased infectivity and HA titers of these viruses exhibited a logarithmic trend as the heat-treatment time increased. The thermostable and thermolabile viruses were examined at different time points by the heat-treatment assay.

### Virus NA Assays

The NA activity of viruses was determined by a fluorescence-based assay using a Neuraminidase Assay Kit (Beyotime Biotechnology, China) according to the manufacturer’s instructions. Briefly, 70 μL of detection buffer and 10 μL of allantoic fluid infected with NDVs were added to 96-well polystyrene plates, followed by the addition of 10 μL Milli-Q water. The solution was then mixed gently for 2 min. Then, 10 μL of NA fluorescent substrates was added to the solution and mixed well. After incubation at 37°C for 30 min, the fluorescence with an excitation wavelength of 322 nm and an emission wavelength of 450 nm was measured by a fluorescence spectrophotometer (BioTek, United States). NA activity is shown as the fluorescence intensity of the samples minus the background values of non-infected allantoic fluid.

### Syncytium Formation Assay

The fusion activities of the NDVs were examined as previously described (Jin et al., 2016; Chi et al., 2019). Briefly, 10^5^ TCID_50_/mL of NDVs were heat-treated at 56°C for 10 min, and then infected with Vero cells for 2 h at 37°C. Then the infected cells supernatant was discarded and kept in the DMEM (Gibco) supplemented with 2% FBS. At 36 h post infection, cell monolayers were washed with PBS for three times and fixed with pre-cooled methanol for 15 min. The cell monolayers were stained with Giemsa solution for syncytia observation under an inverted microscope. Syncytia are cells with three or more nuclei. Fusion activity was calculated from the fusion index, which involved calculating the ratio of the total number of nuclei to the number of cells in which these nuclei were observed. The value obtained from fusion index for the NDVs was expressed as the percentages of the value for the HR09 virus, which was considered to be 100%.

### Statistical Analysis

All statistical analyses were performed using GraphPad Prism Software Version 5.0 (GraphPad Software Inc., San Diego, CA, United States). Measured values are expressed as the mean with standard deviation (SD). Statistically significant differences between different groups were assessed using a *t*-test. A value of *p* < 0.05 was considered significant.

## Results

### Biological Characteristics of the Chimeric NDVs

The role of the F and HN genes in the thermostability of NDV was investigated. Six ICs of chimeric NDVs were constructed; the ORFs of F and/or HN were exchanged between the ICs of the heat-resistant strain HR09 and the thermolabile strain La Sota (Figure 1). The chimeras cHR-La-F, cHR-La-HN, cHR-La-F/HN, cLa-HR-F, cLa-HR-HN, and cLa-HR-F/HN from constructed chimeric ICs were obtained using a reverse genetics system.

**FIGURE 2:**
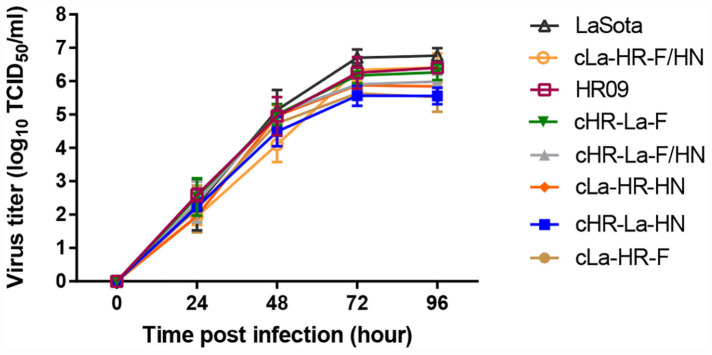
Growth kinetics of NDVs in chicken embryo fibroblasts (CEFs). CEFs were infected (multiplicity of infection = 0.01) with parental and different chimeric viruses. At the indicated time points, infected cells and culture supernatants were harvested for virus titration in CEFs. Mean and standard deviations are shown from three independent experiments.

The pathogenicity of chimeric viruses was investigated. The chimeric NDVs were performed by MDT assay in 10-day-old SPF chicken embryos (Table 2). Of the two parental viruses, HR09 (60.8 ± 2.6 h) was classified as a virulent strain, and La Sota (109 ± 2.6 h) was classified as an avirulent strain. The MDT values of the three chimeric viruses, cHR-La-HN (67.8 ± 5.5 h), cLa-HR-F (77.3 ± 3.8 h) and cLa-HR-F/HN (73.5 ± 3.7 h), possessed velogenic properties, whereas the other chimeric viruses retained the lentogenic pathotype. However, there were no significant differences in virus titers among these chimeras in SPF chicken embryos, and the EID_50_/0.1 mL values of chimeric viruses except cLa-HR-F were almost more than 8.0 log_10_. The growth kinetics of parental and chimeric NDVs were evaluated in CEFs (Figure 2). The parental and chimeric NDVs possessed high titers at 96 h post infection and the difference is not significant (*p* > 0.05).

**TABLE 2:** Pathogenicity and growth titer of chimeric NDVs.

Strain	Pathogenicity	Virus titer	
		
	MDT (h)	EID_50_/0.1 mL (log_10_)	HA (log_2_)
HR09	60.82.6	8.5	11.0
cHR-La-F	99.74.8^b^	8.1	8.0
cHR-La-HN	67.85.5	8.3	10.0
cHR-La-F/HN	1036.0^b^	8.5	9.0
La Sota	1092.6^c^	8.7	11.0
cLa-HR-F	77.33.8^a^	7.9	9.0
cLa-HR-HN	1031.2^b^	8.5	10.0
cLa-HR-F/HN	73.53.7^a^	8.3	9.0

*P-values are represented as follow: ^*a*^ < 0.05; ^*b*^ < 0.01; ^*c*^ < 0.001, compared to HR09 virus. Means and standard deviations are shown for three independent experiments.*

### HN Protein Is the Crucial Thermostable Determinant

**FIGURE 3:**
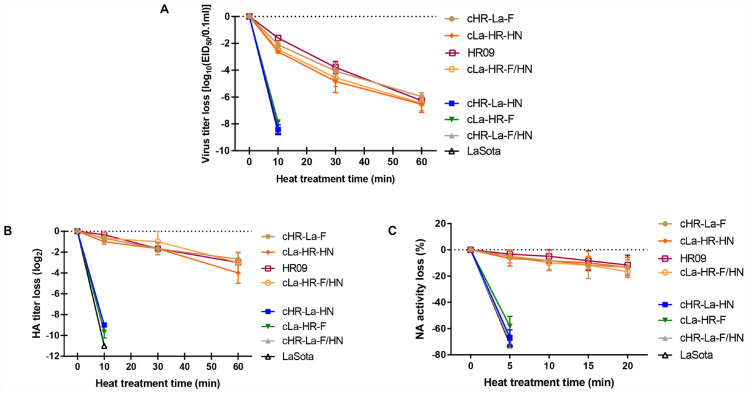
The thermostability and neuraminidase (NA) activity tests for chimeric NDVs. Heat-inactivation kinetics of infectivity of parental and chimeric NDVs were determined at 56°C by performing an EID_50_ **(A)**, HA activity **(B)**, and NA activity **(C)** assay. Values are the average of three independent tests.

To compare the thermostability of chimeric or parental viruses, all the chimeric or parental viruses were treated at 56°C for the indicated time and then examined by performing an EID_50_ assay (Figure 3A). The cHR-La-HN, cLa-HR-F, cHR-La-F/HN and La Sota almost completely loss the infection at a mean time of 10 min. However, the infectivity titers of cHR-La-F, cLa-HR-HN, and cLa-HR-F/HN was about 10^4^ EID_50_/0.1 mL after 30 min, similar to that of the HR09 strain. According to the criteria for the thermostability of the NDV strains, chimeric viruses containing the HN gene of the HR09 strain, including cHR-La-F, cLa-HR-HN, and cLa-HR-F/HN, were thermostable viruses, whereas those containing the HN gene of the La Sota strain, including cHR-La-HN, cLa-HR-F, and cHR-La-F/HN, were thermolabile viruses. These data indicate that the crucial thermostable determinants of NDV are located within the HN gene.

The differences in HA and NA activities of NDVs after 56°C heat treatment were investigated. The parental and chimeric viruses were evaluated *in vitro* by performing the HA and NA activity tests. The HA titer of the thermolabile NDVs containing the HN gene of La Sota decreased sharply, by more than 9.0 log_2_ at a mean time of ≤10 min, whereas the HA titers of the thermostable NDVs encoding the HR09 HN gene declined approximately by 4.0 log_2_ in 60 min (Figure 3B). Similar results were obtained from the NA activity assay, and the NA activity loss of NDVs containing the La Sota HN gene decreased more than 50% in 5 min, however, viruses containing the HR09 HN gene lost less than 20% NA activity in 20 min (Figure 3C). These results demonstrated that the HA and NA activities of the HR09 HN protein were more thermostable than those of the La Sota HN protein.

### Identification of Thermal Sensitive Regions by Temperature Dependent Molecular Dynamics Simulations

**FIGURE 4:**
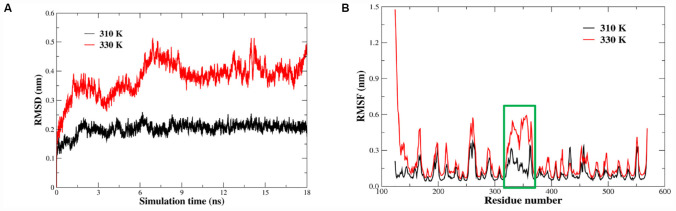
Molecular dynamics simulation results for the HN protein of the strain La Sota over 18 ns at different temperatures. **(A)** The root mean square deviation (RMSD) curves of HN protein during the simulations at different temperatures where black is 310 K and red is 330 K. **(B)** The root mean square fluctuation (RMSF) calculation is based on the C_α_ atoms of HN protein at 310 K and 330 K, respectively. Significant differences are indicated by the green box.

The stability and dynamics of the HN protein of La Sota strain at different temperature have been assessed including the variations of the RMSD and RMSF. Our analysis relies on 18 ns of the molecular dynamics trajectory at temperatures of 310 K and 330 k, respectively. The RMSD values of the backbone atoms for HN protein are shown in Figure 4A, it is almost stable around of value 0.2 nm during the simulation at 310 K. At 330 K, the RMSD fluctuation increases up to 0.45 nm at approximately 7 ns. However, the conformation of HN protein system become stable during the MD simulation after 9 ns. Average RMSF values of amino acid residues in the MD simulation are usually considered as the criterion for structural flexibility of the protein system. We further assessed the RMSF of all residues to quantify the local variation in the coordinates of atom (Figure 4B). The result showed that the RMSF values in most regions of the HN protein of the thermolabile strain merely showed slight fluctuations with increasing temperature, and the region of 315–375 amino acids (green box) exhibited steeply increased at 330 K, indicating that this is the thermally sensitive region of structure.

### Recovery of the Mutated NDVs and Biological Characteristics

**FIGURE 5:**
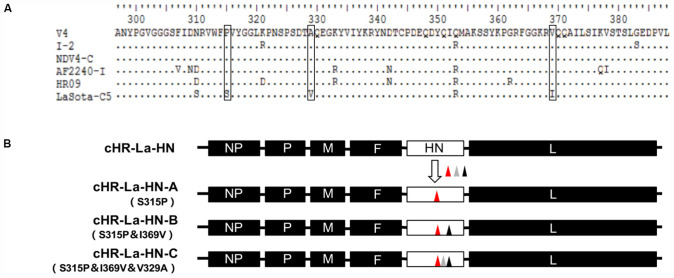
Schematic representation showing the construction of mutant NDVs. **(A)** Multiple amino acid sequence alignment of HN proteins. The black box denotes the point variation in identity between thermostable and thermolabile strains. **(B)** Schematic representation of the construction of mutant viruses. The mutated ICs containing the S315P, I369V and/or V329A substitution(s) were designated as cHR-La-HN-A, cHR-La-HN-B, and cHR-La-HN-C, respectively, according to the mutation sites. The triangles with different colors indicates the different mutation sites.

To further clarify the trait of the thermally sensitive region, we conducted an amino acid sequence alignment of HN proteins of thermostable and thermolabile strains. The thermostable strains contained amino acid substitutions of S315P, V329A, and I369V compared with the thermolabile strains (Figure 5A). To identify whether the three amino acid substitutions could affect viral thermostability, three mutant viruses based on thermolabile cHR-La-HN were generated by single, double or triple amino acid substitutions at positions 315, 369, and 329, and the strains cHR-La-HN-A, cHR-La-HN-B, and cHR-La-HN-C were successfully obtained (Figure 5B).

**FIGURE 6:**
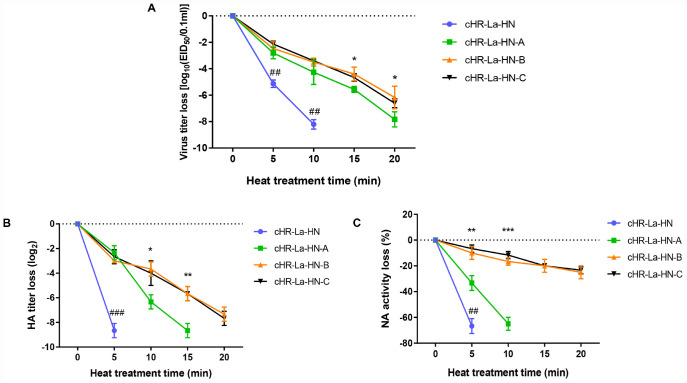
The thermostability and neuraminidase (NA) activity tests of the mutant viruses. Heat-inactivation kinetics of infectivity of different viruses were evaluated by EID_50_ assay **(A)** at 56°C. *p*-values are represented as follow: * < 0.05; ^##^ < 0.01, compared to cHR-La-HN-A. Heat-inactivation kinetics of HA activity **(B)** and NA activity **(C)** of different viruses were determined by HA and NA tests at 56°C. *p*-values are represented as follow: * < 0.05; ** < 0.01; *** < 0.001; ^###^ < 0.001, compared to cHR-La-HN-A. Means and standard deviations are shown for three independent experiments.

The differences in biological characteristics between the chimeric cHR-La-HN and mutant viruses were investigated. The pathogenicity of three mutant viruses was determined by MDT assay in SPF chicken embryos. The results showed that three mutant viruses remained virulent, and the MDT values of three mutant viruses showed no significant difference from those of the chimeric cHR-La-HN (*p* > 0.05), but significant higher than those of HR09 (*p* < 0.05). In addition, there were no significant differences in virus titers between the mutant viruses and chimeric cHR-La-HN; and the chicken embryos infectivity titers were ≥8.3 log_10_ EID_50_/0.1 mL, and the HA titer was 10.0 log_2_, respectively (Table 3).

**TABLE 3:** Pathogenicity and growth titer of mutant NDVs.

Strain	Pathogenicity	Virus titer	
		
	MDT (h)	EID_50_/0.1 mL (log_10_)	HA (log_2_)
cHR-La-HN-A	71.7 ± 2.1	8.3	10.0
cHR-La-HN-B	72.5 ± 1.4	8.5	10.0
cHR-La-HN-C	70.1 ± 4.5	8.3	10.0

### The NDV Mutants Are More Thermostable Than the Chimeric Virus

To investigate the difference in thermostability between chimeric virus and mutant viruses, heat resistance was evaluated by performing an EID_50_ assay after heat treatment at 56°C (Figure 6A). Within 10 min, the titers of cHR-La-HN decreased strikingly by approximately 8.0 log_10_, which was significantly higher than the decrease in the titers of the mutant viruses (*p* < 0.01), with a titer loss of 4.0 log_10_. Among the three mutant viruses, the titer loss of cHR-La-HN-A was higher than that of cHR-La-HN-B and cHR-La-HN-C within 20 min (*p* < 0.05). Additionally, there was no statistically significant difference in the titer loss between cHR-La-HN-B and cHR-La-HN-C at the same heat treatment time.

To determine the differences in HA and NA activities between the chimeric and mutant viruses at 56°C, the chimeric and mutant viruses were examined by performing HA and NA activity assays after heat treatment for 20 min at 56°C. The HA titer of cHR-La-HN declined sharply, by more than 8.0 log_2_ in 5 min, whereas the HA titer of three mutant viruses decreased approximately by 3.0 log_2_ at the same time. In addition, the HA titer loss of cHR-La-HN-A was much greater than that of cHR-La-HN-B and cHR-La-HN-C at 15 min (*p* < 0.05). There was no statistically significant difference in the HA titer loss between cHR-La-HN-B and cHR-La-HN-C at the same heat treatment time (Figure 6B). Similar results were obtained from the NA activity assay, and the NA activity loss of the three mutant viruses was much less than that of cHR-La-HN in 5 min (*p* < 0.01). Moreover, the NA activity of cHR-La-HN-B was similar to that of cHR-La-HN-C, and the activities of both cHR-La-HN-B and cHR-La-HN-C were much higher than that of cHR-La-HN-A (Figure 6C).

### The Mutant Viruses Display Higher Fusion Activity Than the Chimeric cHR-La-HN

**FIGURE 7:**
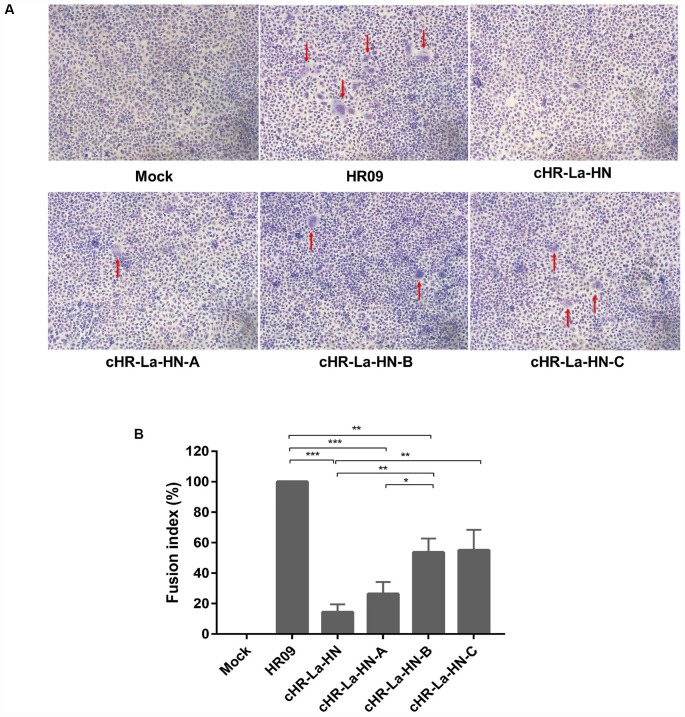
Syncytium formation induced by different NDVs. **(A)** Vero cells were infected with different NDVs that were heat-treated at 56°C for 10 min. Different groups of cells were stained with Giemsa solution at 36 h post-infection. Images were immediately acquired under an inverted microscope at × 100 magnification. The syncytia formation were indicated by red arrows. **(B)** The fusion index values for the different NDVs were calculated as the ratio of the total number of nuclei to the number of cells in which the nuclei were observed. All values are expressed relative to the value for HR09 (100%). Means and standard deviations are shown for three independent experiments. *p*-values are represented as follow: * < 0.05; ** < 0.01; *** < 0.001.

In order to explore the effects of the mutant viruses and chimeric virus on their fusogenic activity after heat treatment at 56°C, the syncytium formation was observed and fusion index assay was performed in Vero cells (Figure 7). The HR09 still caused the severe syncytia after heat treatment 10 min at 56°C, however, there was no significant syncytia formation induced by the cHR-La-HN at the same conditions. It was found that more syncytia were formed by cHR-La-HN-B and cHR-La-HN-C compared with that produced by cHR-La-HN-A (Figure 7A). Additionally, quantification of the fusogenic abilities of the mutants and chimeric virus (fusion index compared with HR09) showed that cHR-La-HN, cHR-La-HN-A, cHR-La-HN-B, and cHR-La-HN-C had significantly decreased the fusion activities (14, 26, 53, and 55%, respectively) (Figure 7B). It should be noted that the fusion index of the cHR-La-HN-B and cHR-La-HN-C were significant higher than those of the cHR-La-HN (*p* < 0.01). These results suggested that when the HN gene of thermostable HR09 was replaced by thermolabile La Sota, the fusion activity of chimeric virus have been decreased rapidly after heat treatment at 56°C. The mutant viruses possessed higher fusion activity than the chimeric virus under the same heat treatment condition.

### Residues Ser315 and Ile369 Are Close to the Receptor Binding Sites

**FIGURE 8:**
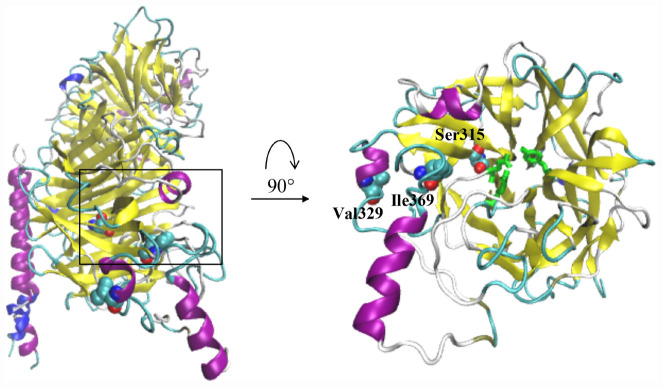
The 3D structure of the HN protein was generated by the homology model of the crystal structure of the HN ectodomain of the NDV/Victoria/32 (PDB ID code: 3t1e.1.A). Schematic drawing of the HN dimer showing the location of the residues Ser315, Ile369 and Val329. The main sialic acid receptor binding sites (Arg416, Glu401, and Tyr526) are indicated in green.

To analyze the location of the amino acids at positions 315, 329, and 369, the structure of the HN protein of the La Sota strain was analyzed, and the locations of Ser315, Val329, and Ile369 are indicated in Figure 8. The Ser315 residue is located in the sheet structure, and the Ile369 residue is located at the junction of the β-sheet and loop. Both of these residues are also adjacent to the major sialic acid binding sites, which are labeled in green (Arg416, Glu401, and Tyr526).

## Discussion

As an effective molecular tool, reverse genetics systems have enabled the role of viral proteins in viral replication and virulence to be determined by genetic approaches (Kim et al., 2011). In this study, to investigate the relationship between the F and HN genes and viral thermostability, we successfully constructed six chimeric viruses by reverse genetics. The results showed that replacement of the HN gene in the thermolabile strain La Sota with the HN gene from the heat-resistant strain HR09 resulted in significantly enhanced thermostability (Figure 3). In contrast, the replacement of the HN gene in the HR09 strain with that in the La Sota strain led to reduced thermostability. These data show that the HN gene plays an important role in the thermostability of virulent NDVs. These results are consistent with those of previous studies (Wen et al., 2016; Liu et al., 2019). Liu et al. (2019) also reported that F gene, especially amino acid sequence of cleavage sites could affect the thermostability of NDV. Therefore we think that both F and HN genes are associated with viral thermostability, of which HN gene is most important determinant according to our results. In addition, F gene is critically involved in determining viral virulence, as shown in this study in Table 2. All chimeric viruses containing F gene of the virulent HR09 possessed virulent pathotype, and the MDT values of them were lower than those of chimeric viruses containing F gene of the La Sota (*p* < 0.05). Although, it is well-known that the HN gene is responsible for the replication and pathogenicity of NDV, the role of the HN gene in viral thermostability has not been well understood. Sequence alignment of the HN protein from different thermostable strains showed that the HN proteins of genotype I strains V4 and I-2 have 616 and 578 amino acids, respectively, whereas the HN protein of the HR09 strain has 572 amino acids, which is less than that of AF2240-I (583 amino acids) (Kattenbelt et al., 2006; Murulitharan et al., 2013). Whether these differences in the amino acid length of the HN protein affect viral thermostability remain to be further studied.

MD simulation has widely been used to explore the thermal-stable mechanism of industry enzymes (Purmonen et al., 2007; Rouhani et al., 2018). By calculating the RMSF values of backbone atoms, thermally sensitive regions or structural flexibility were identified (Cunha et al., 2015; Unal et al., 2019). In this study, we investigated the changes in the RMSF value of the HN protein by changing the temperature to 310 and 330 K. We found that residues 315–375 of the HN protein in the La Sota strain caused the major instability of the structure. We also compared the amino acid sequences of the HN proteins in thermostable and thermolabile strains. There were significant differences in three amino acids at the unstable regions of the HN proteins. Whether proline substitution at position 315 would affect the thermostability of NDV was determined. We used a site-directed mutation method to replace serine with proline based on thermolabile cHR-La-HN strain. The results showed that replacing Ser315 with Pro significantly enhanced the thermostability of NDV. It was proposed that proline residues restrict backbone bond rotation due to their pyrrolidine rings, thereby decreasing the conformational entropy during protein unfolding and consequently leading to protein stabilization (Prajapati et al., 2007). Introduction of proline residues increases protein stability and has been successfully reported in industrial enzymes. In particular, proline residues at specific sites, such as β-turns or loop regions, are more effective for protein thermostability (Tian et al., 2010; Bustamante et al., 2014).

The sialic acid binding sites of the HN protein play a key role in the attachment of viruses to cells and are usually conserved across isolates irrespective of their thermostability phenotypes (Yuan et al., 2011). Residues Arg416 as well as Glu401 and Tyr526 are essential for receptor binding; these residues also meditate fusion promotion and neuraminidase activity (Connaris et al., 2002; Mahon et al., 2011). Here, we examined the effect of mutation S315P, I369V, and V329I of the HN protein on the HA and NA activity. The results showed that the mutant viruses possessed the higher HA and NA activity than the parental virus after heat treatment at 56°C (Figure 6). However, there was no difference in the HA or NA activity or thermostability of the V329I mutation. Compared with the chimeric cHR-La-HN, the mutant viruses were proved to increase the fusogenic capacity under the 56°C heat treatment condition (Figure 7). By analyzing the HN protein 3D structure, we found that in comparison with the Val329, both Ser315 and Ile369 are closer to sialic acid binding sites in spatial structure (Figure 8), which may contribute to stabilizing the function of fusion and NA activity. Therefore, we speculated that when the mutant viruses were subjected to heat treatment at 56°C, viral HA, fusion and NA activity could be retained. This allowed the initiation of viral infection and replication.

In summary, our study reveals that the HN protein is the major determinant of NDV thermostable. Furthermore, the mutations of S315P and I369V in the HN protein could contribute to enhancing viral thermostability and HA and NA activity. These findings have important implications for understanding the thermostable mechanism of NDV and play a positive role in thermostable NDV vaccine development.

## Data Availability Statement

The datasets presented in this study can be found in online repositories. The names of the repository/repositories and accession number(s) can be found below: https://www.ncbi.nlm.nih.gov/genbank/, JX524203.1; https://www.ncbi.nlm.nih.gov/genbank/, AY935499.2; https://www.ncbi.nlm.nih.gov/genbank/, JX443519; https://www.ncbi.nlm.nih.gov/genbank/, MF285077; https://www.ncbi.nlm.nih.gov/genbank/, JX012096.2; https://www.ncbi.nlm.nih.gov/genbank/, KC844235.1.

## Author Contributions

YC, XZ, and YW carried out the study design. BR, CM, and PD performed the experiment and data analysis. BR wrote the manuscript. MG, CZ, and YW contributed to the revision of the manuscript. All authors read and approved the final manuscript.

## Conflict of Interest

The authors declare that the research was conducted in the absence of any commercial or financial relationships that could be construed as a potential conflict of interest.

## Footnotes

1 https://swissmodel.expasy.org
