# Supplementary material for: Residues 315 and 369 in HN Protein Contribute to the Thermostability of Newcastle Disease Virus
Source: Front Microbiol. 2020 Sep 22;11:560482. doi: 10.3389/fmicb.2020.560482 (PMC7536312; doi:10.3389/fmicb.2020.560482)
Supplement: Supplementary file 1 [file Data_Sheet_1.pdf]

The stability and dynamics of the HN protein of the heat stable strain HR09 at different temperature have been assessed including the variations of the RMSD and RMSF. As can be seen in **Supplemental Figure 1A**, the RMSD curves tend to the stability, with the increase of simulation times (ns). The RMSD curve stability indicates the reliability of simulation system. Analysis result shows that the conformation of HN protein system become stable after 9 ns of MD simulation time. In this study, our analysis relies on 18 ns of the molecular dynamics trajectory at temperatures of 310 K and 330 k, respectively. RMSF values reflect fluctuation at individual residues – a higher RMSF value indicates less stability. The RMSF result shows that the HN protein structure of the heat stable strain HR09 becomes more unstable as the temperature increases **(Supplemental Figure 1B**).

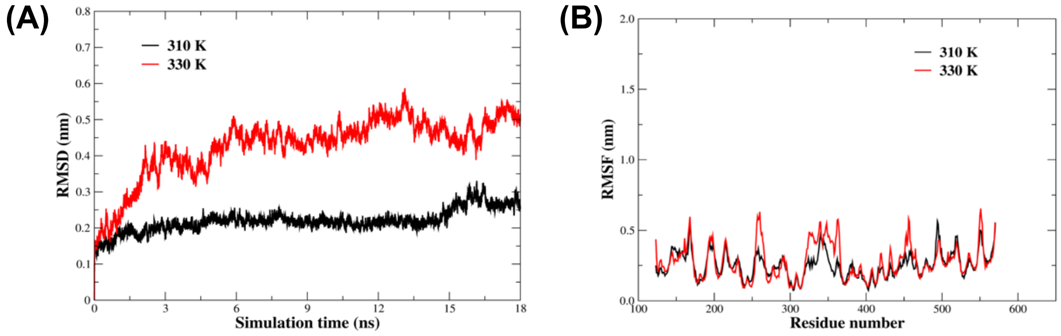

Supplemental Figure 1 The RMSD and RMSF analyses of the HN protein in HR09 strain. (A) The time-dependent RMSD values of HN protein at different temperatures (310 K and 330 K); **(B)** RMSF values of HN protein in the heat stable strain HR09.

The stability and dynamics of the HN protein of the mutant strains at 310 K and 330 K have been assessed including the variations of the RMSD and RMSF. As can be seen in **Supplemental Figure 2A**, the RMSD curves tend to the balance, with the increase of simulation times (ns). In this study, our analysis relies on 10 ns of the molecular dynamics trajectory at different temperatures. Molecular dynamics simulation results are shown in following Supplemental Figure 2, the RMSF values of the two-point (**Supplemental Figure 2C**) and three-point mutant strains (**Supplemental Figure 2D**) are lower than that of the single mutant strain (**Supplemental Figure 2B**). The results indicate that HN protein structure of the single mutant strain is more unstable than those of the two-point mutant and three-point mutant strains at 330 K.

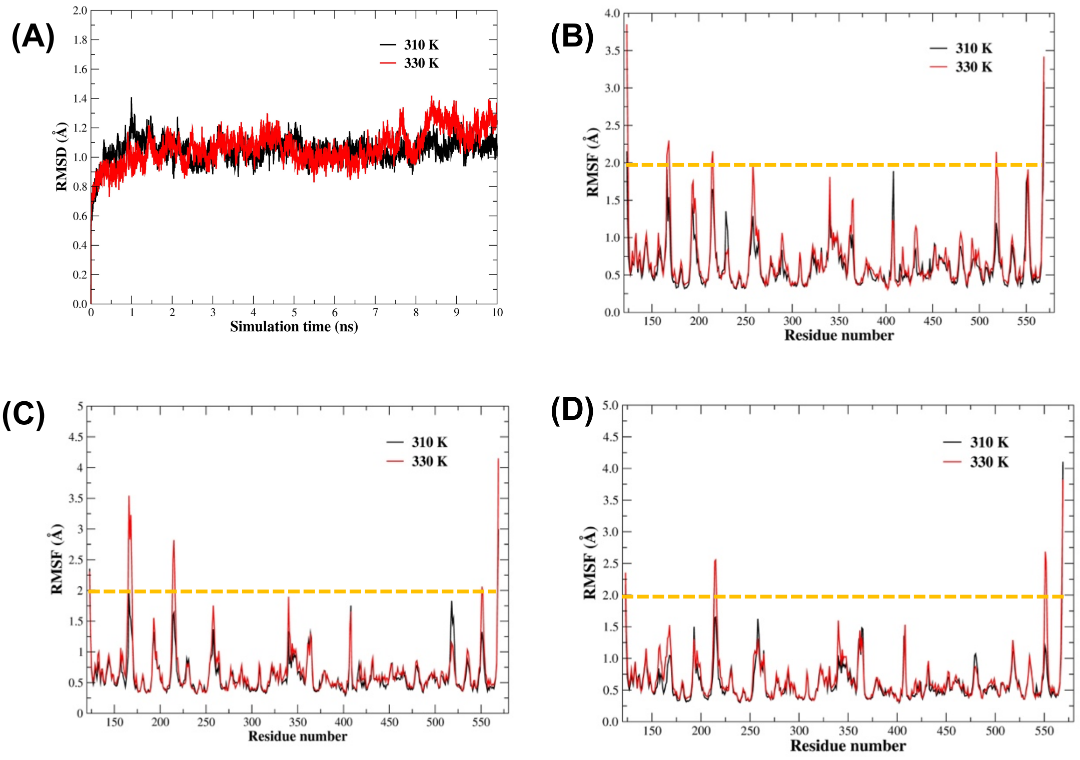

Supplemental Figure 2 The RMSD and RMSF analyses of the HN proteins in mutant strains. (A) The time-dependent RMSD values of HN protein at different temperatures (310K and 330K); **(B)** RMSF values of HN protein in cHR-La-HN-315P strain; **(C)** RMSF values of HN protein in cHR-La-HN-315P/369V strain; **(D)** RMSF values of HN protein in cHR-La-HN-315P/369V/329A strain. (1 Å=0.1 nm)
